# If the data contradict the theory, throw out the data: Nicotine addiction in the 2010 report of the Surgeon General

**DOI:** 10.1186/1477-7517-8-12

**Published:** 2011-05-19

**Authors:** Hanan Frenk, Reuven Dar

**Affiliations:** 1 Department of Psychology, Tel Aviv University, Ramat Aviv 69978, Israel; 2 The School of Behavioral Sciences, The Academic College of Tel Aviv-Yafo, Tel Aviv, Israel

**Keywords:** tobacco smoking, nicotine dependence, Surgeon General, addiction

## Abstract

The reports of US Surgeon General on smoking are considered the authoritative statement on the scientific state of the art in this field. The previous report on nicotine addiction published in 1988 is one of the most cited references in scientific articles on smoking and often the only citation provided for specific statements of facts regarding nicotine addiction. In this commentary we review the chapter on nicotine addiction presented in the recent report of the Surgeon General. We show that the nicotine addiction model presented in this chapter, which closely resembles its 22 years old predecessor, could only be sustained by systematically ignoring all contradictory evidence. As a result, the present SG's chapter on nicotine addiction, which purportedly "documents how nicotine compares with heroin and cocaine in its hold on users and its effects on the brain," is remarkably biased and misleading.

## Background

The reports of US Surgeon General on smoking are considered the authoritative statement on the scientific state of the art in this field. The previous report [1] is one of the most cited references in scientific articles on smoking and is often the only citation provided for specific statements of facts regarding smoking. As such, one would expect this official report to present an updated and carefully balanced view of the research on smoking. At least as concerns the issue of nicotine addiction, however, the latest report [2] fails to fulfill this mission. The new report adheres to the former one of 1988 [1] in equating smoking with nicotine addiction. It reiterates the three major conclusions of the 1988 report, namely that (1) cigarettes and other forms of tobacco are addicting, (2) nicotine is the drug in tobacco that causes addiction and (3) the pharmacologic and behavioral processes that determine tobacco addiction are similar to those that determine addiction to drugs such as heroin and cocaine. Consequently, the terms "tobacco addiction" and "nicotine addiction" are used interchangeably starting on the first page of Chapter 4, which purports to provide the current scientific knowledge regarding nicotine addiction. In the present commentary we address the model of nicotine addiction presented in Chapter 4 of the report. Specifically, we challenge conclusion (2) which states that "nicotine is the drug that causes addiction". We will show that this model could only be sustained by systematically ignoring all contradictory evidence. As a result, the present SG's chapter on nicotine addiction, which purportedly "documents how nicotine compares with heroin and cocaine in its hold on users and its effects on the brain," is remarkably biased and misleading.

How does nicotine cause addiction, according to the authors of the report [1] (references in this citation are omitted)? "The factors that may contribute to addictive behaviors include (1) neuroadaptations that occur with the persistent use of nicotine (e.g., tolerance), (2) withdrawal symptoms experienced when intake of the drug is stopped, and (3) the effects of nicotine that reinforce dependence. The primary reinforcing effects can entail the rewarding (psychoactive or psychostimulant) effects of nicotine (positive reinforcement) and/or the alleviation of aversive or negative states or stimuli--for example, relief from withdrawal symptoms (negative reinforcement). Nicotine may also enhance the reinforcing values of other reinforcers or stimuli, which may also contribute to its reinforcing effects (p.116)".

Thus, the SG's report asserts that nicotine is a primary positive reinforcer and that repeated nicotine administration causes neurobiologic adaptation, which results in tolerance to the effects of nicotine. In the absence of nicotine, a withdrawal syndrome ensues that is alleviated by nicotine and hence makes the drug a negative reinforcer. This model is identical to the model that accounts for addiction to opiates and to other drugs such as alcohol and barbiturates. In the case of nicotine, however, the evidence for the SG's model of addiction is much weaker than the authors of the report portray it to be. Below, we review the principal tenets of the nicotine addiction model presented in the SG's report and examine their empirical status. As we shall show below, the conclusions summarized in the preceding paragraph are invalidated by (a) selectively presenting evidence that supports these conclusions while ignoring evidence that contradicts them, (b) presenting evidence that does not pass criteria for modern science and was discarded by contributors to the report themselves in the recent past, and (c) stating that evidence exists where, in fact, it does not.

### Reinforcement

Is nicotine a primary reinforcer, as claimed by the SG's report? This question has been extensively studied both in animal and in human subjects. Regarding animal studies, the authors of the report [1] state: (p. 111; the reference format has been changed to that of the present journal): "Earlier studies that examined a wide range of animal species have shown that nicotine alone can lead to self administration in preference to an inert control substance [1,3-6])." We have critiqued the animal nicotine self-administration studies in the past [7,8] and the complexity of the relevant issues makes it impossible to repeat the analysis in the context of this commentary. Briefly, most of the studies reviewed by the SG are methodologically flawed and their results confounded by (a) training the animals to lever press for food on an "active" lever and then switching them to i.v. nicotine for pressing the same lever while keeping the animals food-deprived [9]; (b) confounding nicotine effects with those of the concurrent visual stimuli, which are reinforcing by themselves [10]; (c) failing to use adequate controls for the activating properties of nicotine which have been demonstrated in this paradigm [11], (d) eliminating uncooperative animals from the results [12], (e) not using statistics [13] and more. Recent studies [14] that have avoided the pitfalls of the studies cited by this report show nicotine to be at best a very weak reinforcer. For example, in Sorge et al.'s study, the number of presses on the nicotine-delivering lever was extremely low - 3 times per hour - and there was no increase in pressing rate over 15 2 hr sessions. Such findings are inconsistent with the view that nicotine alone can drive a persistent habit such as smoking and surely cannot support the comparison made by the SG between nicotine and drugs such as cocaine or heroin. In fact, one would be hard pressed nowadays to find such preposterous statements regarding the reinforcing power of nicotine outside the SG report.

Putting aside the debate about nicotine's reinforcing properties in animals, it is uncontroversial that in order to drive smoking, nicotine must be reinforcing to humans. We shall therefore focus the remaining of this commentary on the evidence for nicotine addiction in human smokers, beginning with self administration studies. This is what the present report claims in this regard: "Humans have also demonstrated a preference for nicotine over a control substance in studies examining intravenous administration [15,16], nasal administration [17], and use of medicinal gum [18]." This statement is a misrepresentation of the facts. Our review of all nicotine self administration laboratory studies published up to 7 years ago [19] found that *none of them *demonstrated nicotine self-administration in smokers. Both smokers and non-smokers did not show any preference for nicotine over placebo in any of these studies, including in a series of six reports of overnight abstinent smokers having access to nicotine nasal spray, a rapidly absorbed form of nicotine [20-25]. The studies that claimed to have demonstrated self-administration in smokers were invalidated by choosing participants who were illicit drug users [15,16,26], absence of statistics [15,26] or insufficient control for expectations [27] (for critique see [28]). As is the general rule in this chapter of the SG's report, its authors chose to cite few supporting studies (who happen to be mostly their own) and to ignore the great majority of studies that provide compelling evidence against their favored thesis. This is particularly striking considering that one of the contributing editors and cited authors has also acknowledged in 2004 that "[nicotine] has not been clearly shown to maintain intravenous self-administration levels above vehicle placebo levels in humans [16], p. 134."

What about the studies that are cited by the report as showing nicotine self-administration in smokers [17,18] and were not included in our review [29]? Neither of these studies was designed to test whether nicotine was reinforcing to smokers and indeed neither constitutes an adequate test of this hypothesis. First, both studies were conducted with participants who declared a wish to quit smoking. This violates a basic methodological rule in smoking research that the effects of nicotine per se cannot be assessed in participants wishing to quit because of the confounding effects of beliefs and expectations regarding nicotine in such participants. Accordingly, studies that aim to examine the effects of nicotine in smokers explicitly seek participants who declare no intent to quit in the foreseeable future [30]. Second, in these studies participants were not presented with a choice of administering either nicotine or placebo but were assigned to receive either nicotine or placebo. Consequently, "preference" for nicotine over placebo could not really be determined in either of these studies. Opting to present these two studies as evidence for nicotine self administration in smokers and to ignore the gamut of adequately designed studies that did not find any preference for nicotine over placebo demonstrates a disturbing bias by the authors of the SG report.

Another example of the same bias is the way in which a study by Perkins et al. [20] is presented in the SG report. The authors of the report refer to it as follows (p. 120): "The choice of nicotine nasal spray instead of a placebo nasal spray increases with smoking abstinence [20]." This sentence follows immediately after the statement that "Nicotine alone, isolated from tobacco smoke, is reinforcing in humans" giving the impression that it at least consistent with that statement, if not providing further support for it. In fact, what Perkins et al. [20] found was that smokers who were abstinent from smoking prior to the experiment self-administered more nicotine nasal spray than when they were not. However, even those abstinent smokers did not show any preference for nicotine over placebo; both were self-administered equally, each in 50% of the trials. Moreover, when participants were not abstinent, nicotine was actually *aversive*: participants chose to self-administer placebo over nicotine in 70% of the trials. Clearly, these results cannot be taken as supporting evidence for nicotine self-administration in humans.

As further evidence for nicotine reinforcement in humans, the SG reports states that "if levels of nicotine in the body are altered, smokers tend to compensate or titrate their dose by (1) smoking more if the levels of nicotine are reduced or blocked by a nicotinic receptor antagonist or (2) smoking less if exogenous nicotine or higher levels of nicotine are administered [1,31,32]". In regard to point (1), it has been well documented that when smokers are switched to cigarettes with lower nicotine yield they indeed "compensate" by smoking more. But is this compensation really due to reduction in nicotine intake? The objective answer is "probably not." In the vast majority of the experiments in which smokers were switched to cigarettes with lower nicotine yield there was no attempt to separate the effects of nicotine and tar. This is a serious omission considering that the correlation between nicotine and tar yields in commercial cigarettes is .90 [33,34], so that reducing nicotine yield in cigarettes means also reducing tar yield. Therefore, attributing the increased smoking in such studies to reduction in nicotine rather than in tar yield requires a big leap of faith. This leap is unjustified considering that smoking pleasure is determined to a large extent by sensations in the respiratory tract that accompany smoke inhalation and are caused to a large extent by tar [35]. Moreover, there is some evidence that certain non-nicotine constituents of tar may have central actions in brain areas linked to reinforcement. In fact, Sutton et al. [36] found that tar yield predicted puffing patterns (and hence blood levels of nicotine) far better than does nicotine, a finding that was confirmed by several other studies [37-39].

More generally, the present report seems to brush aside the growing body of evidence for the crucial effect of non-nicotine factors in smoking. The importance of the sensory rewards associated with smoking has been documented for decades. More recently, studies with de-nicotinized tobacco have shown conclusively that such factors determine smoking behavior at least as much as nicotine. Smokers readily smoke de-nicotinized cigarettes [40] and there is no decay in the rate of smoking that would be expected if the motivation for smoking was nicotine. In the same vein, de-nicotinized cigarettes are as effective as regular cigarettes, and more than nicotine in any other delivery mode, in relieving withdrawal and craving [41-44]. A particularly compelling demonstration of the reinforcing effects of de-nicotinized smoke in comparison to nicotine was provided by a recent study that allowed smokers to make concurrent choices between IV nicotine, IV placebo, de-nicotinized smoke puffs and sham puffs. This study found that smokers, following 12 hours abstinence, overwhelmingly preferred to self-administer de-nicotinized smoke over IV nicotine [44].

While smokers tend to prefer regular to de-nicotinized tobacco, this small difference is probably not due to the psychoactive effects of nicotine but to its contribution to the sensory impact of smoke through its peripheral receptors in the airways [45-47]. A particularly elegant test of this hypothesis was reported in a study in which participants took a single puff from either regular or de-nicotinized tobacco and had to rate its rewarding effects within 7 seconds of inhalation, which is before nicotine can reach the brain [48]. The authors found that nicotinized puffs were rated as more rewarding than de-nicotinized puffs and that the extent to which nicotine elicited reward was directly correlated with the extent to which nicotine elicited airway sensations. These peripheral effects of nicotine can fully account for the other finding noted in point (1), namely that smokers smoke more following administration of a nicotinic receptor antagonist. As mecamylamine, the nicotine antagonists used in the studies cited in this report, blocks the peripheral as well as the central effects of nicotine, smokers would be motivated to increase their level of smoking to compensate for the loss of airway sensations.

What about the finding noted in point (2), that smokers smoke less if exogenous nicotine or higher levels of nicotine are administered? The authors of the report ignore an alternative interpretation, which was termed "parmacodynamic satiation" [49]. Gori and Lynch observed that a ceiling in plasma nicotine and cotinine levels was reached when smokers consumed about 20 cigarettes per day, which was not significantly exceeded even when smokers consumed up to 60 cigarettes per day. This ceiling seems to be absolute, as others have shown the same phenomenon [50] and the average number of cigarettes smoked in England [51] and the USA [1] before smoking restrictions were imposed coincides approximately with the number of cigarettes needed to reach pharmacodynamic satiation. Note that in this respect, according to Gori and Lynch [49], nicotine actually *limits *smoking. Interestingly, a very recent article in *Nature *supports this hypothesis [52]: it suggests that nicotine controls smoking by triggering an inhibitory motivational signal that acts to limit nicotine intake. Parmacodynamic satiation also provides an alternative explanation to why high levels of exogenous nicotine, administered by nicotine replacement therapy (NRT), can reduce smoking. According to this account, NRTs do not satisfy the smoker's need for nicotine but bring the smoker nearer to the parmacodynamic satiation level. The same hypothesis can also explain why blocking the effects of nicotine with mecamylamine pretreatment increases the intravenous self-administration of nicotine [53].

### Tolerance to the effects of nicotine

Like its 1988 version, the current SG's report claims that nicotine addiction is driven by the same factors that drive addiction to opiates and alcohol. We have shown above that the major factor in this model, namely the presumed reinforcing effects of nicotine, is not supported by empirical evidence. Another factor that drives nicotine addiction, according to this model, is "neuroadaptations that occur with the persistent use of nicotine (e.g., tolerance)." How does neuroadaptation, and specifically tolerance, contribute to drug addiction? With continued use, tolerance can occur to both the pleasurable and the aversive effects of drugs. It is well documented that tolerance occurs to the aversive effects of nicotine, at least up to a certain point (see preceding section) as noted by the authors of the current report [2]: "... tolerance to the aversive effects of nicotine must occur for adolescents to escalate from to two cigarettes per day to one pack per day... (p. 117)." However, while tolerance to the aversive effects of a drug *allows *the user to use increasing amounts of the drug, it does not *motivate *increased use. In contrast, tolerance to the pleasurable effects of the drug can motivate increased use and facilitate addiction, as users must administer increasing amounts of the substance to obtain the desired effects. This is what happens with opiates, but does it also happen with nicotine?

Tolerance to the pleasurable effects of nicotine requires, of course, that the drug would *have *pleasurable effects. According to the authors of the SG's report (p.117): "Despite methodologic limitations, studies have clearly shown a chronic tolerance for many self-reported responses to nicotine, such as subjective mood. For example, smokers show fewer responses than do non-smokers to the same amount of nicotine, as evidenced by measures of subjective stimulation *that may be viewed as pleasurable, such as arousal, vigor, and a subjective experience often referred to as "head rush" or "buzz," *[italics ours] as well as some experiences that may be viewed as aversive, including tension and nausea [54]".

The phrasing "that may be viewed as pleasurable" suggests that this view is not supported by compelling evidence. Indeed, it is not. Perkins et al. [55] analyzed subjective responses to nicotine, and specifically noted that head rush "was correlated with negative affect in this study (p. 872)." Moreover, Perkins et al. [54], which is cited above as supporting the possibility that head rush is pleasurable, measured the subjective pleasure participants derived from self-administered nicotine nasal spray directly using a Visual Analogue Scale (VAS). The results show that the values, expressed as difference from pre-dose baseline, were all *negative*. This means that the participants in that study derived no pleasure whatsoever from the nicotine. It seems puzzling that such results are interpreted in the SG's report as evidence for tolerance to the pleasurable effects of nicotine.

Or perhaps it is not so puzzling. If the authors of the SG's report wanted to support their assertion that nicotine undergoes tolerance to its pleasurable effects they had to scratch the bottom: we are not aware of *any *compelling evidence that nicotine has pleasurable effects in smokers. A review by Gilbert [56] concluded that "with few exceptions, nicotine has consistently failed to increase pleasantness and euphoria in experimental studies" (p. 114). Our own review [7] found that lumping across various modes of delivery, nicotine was found to be pleasurable for smokers in only 7 out of 22 studies. In a more recent review, Kalman and Smith [57] found that positive mood effects of nicotine appear to be relatively small and subtle. The review concluded that "taken together, the evidence that the subjective effects of nicotine directly mediate its reinforcing effects is quite modest." Prominent exceptions to the failure to demonstrate significant positive subjective effects of nicotine were two laboratory studies by Pomerleau and Pomerleau [58,59]. However, in these experiments participants were *expressly told *to interpret the sensations of rush, buzz, or high as pleasurable. As our survey of smokers [60] showed, these instructions introduce a bias, as smokers actually perceive the sensation of buzz as aversive. This bias proved to be critical: when we replicated the procedure of the two studies [58,59] using the original instructions, nicotine appeared to produce euphoric effects. However, reversing the instructions by telling participants that rush, buzz and high were *unpleasurable *reversed the findings of the original studies and would have led to the conclusion that nicotine is dysphoric to smokers [60].

### Nicotine withdrawal symptoms

Among the factors that contribute to nicotine addiction, as cited above, the SG report lists "withdrawal symptoms experienced when intake of the drug is stopped." The report states (p. 117-118): "In tobacco-dependent smokers, a reliable consequence of abstaining from smoking for more than a few hours is the onset of distress indicated by self-reported behavioral, cognitive, and physiological symptoms and by clinical signs [61-63]. The subjective symptoms of withdrawal are manifested by affective disturbance, including irritability and anger, anxiety, and a depressed mood. The behavioral symptoms include restlessness, sleep disturbance, and an increased appetite, typically assessed by self-reports. Cognitive disturbances usually center on difficulty concentrating [62,63]. [---] Withdrawal symptoms typically emerge within a few hours after the last cigarette is smoked, peak within a few days to one week, and return to precessation baseline levels after two to four weeks [62,63]".

These and related paragraphs can only be sustained by a very selective presentation of the evidence. First, the authors do not provide any evidence that the withdrawal symptoms mentioned are in any way related to decreased nicotine levels. Such evidence is sorely needed, since many appetitive habits that do not involve drugs, such as eating [64,65], gambling [66,67] or surfing the internet [68] are associated with withdrawal and craving levels that are often as powerful as those reported for the most addictive drugs. As smoking combines (and therefore confounds) an appetitive behavioral habit and a drug, withdrawal symptoms and craving for smoking cannot be equated with craving for nicotine.

Second, craving and withdrawal symptoms are often dissociated from actual smoking (nicotine consumption) or from plasma levels of nicotine. For example, religious Jews who do not smoke during the Sabbath [69] reported no craving or withdrawal symptoms on Saturday morning, following an overnight abstinence, but high levels of craving during a workday when they smoked ad lib. Similarly, non-daily smokers reported much higher craving levels on days that they smoked as compared to days that they did not smoke [70]. A study of flight attendants who are banned from smoking during the flight [71] showed that craving was related to the time remaining to the end of the flight more than to the length of abstinence (and presumably of nicotine withdrawal). In the same vein, neural responses to smoking cues in an fMRI study were related to expectancy to smoke more than to abstinence [72]. These findings are inconsistent with the notion that craving and withdrawal symptoms ensue from lack of nicotine.

Third, if withdrawal and craving result from lowered nicotine levels in the brain, we would expect that nicotine made available by Nicotine Replacement Therapies (NRT's) would be completely abolish withdrawal symptoms and craving. Although partial reduction of withdrawal symptoms was reported [73-75] we are not aware of a single study where all withdrawal symptoms and craving were suppressed by nicotine. The partial reduction in withdrawal achieved by NRT could well be the result of the inadequacy of the placebo controls used in the majority, if not all, of these studies. Several laboratory studies using the balanced placebo design demonstrate that smokers' responses to nicotine are determined to a large extent by their beliefs and expectations regarding nicotine [76-78]. A secondary analysis of a large field study of smoking reduction showed that the success of the treatment was associated more with smokers' *beliefs *about whether or not they received nicotine than with whether or not they actually received nicotine [79]. Note that the limited effect that NRTs have on withdrawal and craving has nothing to do with pharmacokinetics such as the speed of delivery: According to the SG's model there should be no withdrawal as long as nicotine receptors are occupied by the ligand.

Fourth, if the craving smokers experience is for nicotine we would expect that de-nicotinized cigarettes would be far less effective in suppressing withdrawal and craving than NRTs. Quite a few experiments show exactly the opposite: de-nicotinized tobacco is typically as effective a regular tobacco [41,43,80-82] and more than nicotine (other than in tobacco) [30] in suppressing craving and withdrawal symptoms. The fact that these results are not mentioned in the current report is yet another omission that demonstrates its biased portrayal of the reality of nicotine research. These findings also show that if nicotine is a negative reinforcer, as the 2010 report of the SG contends [2] (p.116), it is a much weaker reinforcer than denicotinized cigarettes.

### Precipitated withdrawal

Precipitated withdrawal is the occurrence of an acute withdrawal syndrome in dependent organisms, resembling spontaneous withdrawal, by the administration of an antagonist blocking the receptors to which the drug binds. Naloxone, an opiate antagonist, precipitates a withdrawal syndrome in opiate dependent rats and humans that is identical to the spontaneous withdrawal that occurs when drug administration is stopped. If a similar phenomenon could be demonstrated with nicotine in smokers it would certainly substantiate the thesis that nicotine produces physical dependence. But it is not the case.

Nicotine withdrawal in animals is discussed for nearly 3 full pages (p. 131-133). The authors state (p.131; references in this citation are omitted): "One of the first and most widely used measures developed to investigate the neurobiology of the nicotine withdrawal syndrome and nicotine dependence is the frequency of somatic signs reliably observed in rats, but less reliably observed in mice [---]. The most prominent somatic signs in rats are abdominal constrictions (writhes), gasps, ptosis, facial fasciculation, and eyeblinks. These somatic signs are both centrally and peripherally mediated". Specifically in regard to precipitated withdrawal in rats, the report states that "the observation that nAChR antagonists precipitate the behavioral and neurochemical signs of withdrawal in nicotine-dependent rats, but not in controls, suggests that chronic exposure to nicotine induces a compensatory reduction in endogenous cholinergic tone that leads to the nicotine withdrawal syndrome (p. 133)".

The keen reader will immediately notice that the withdrawal symptoms observed in rats, as described above, bear no resemblance to the "withdrawal syndrome" attributed to abstinent human smokers (see Nicotine Withdrawal Symptoms above). Indeed, there is no reason to believe that the nicotine withdrawal symptoms described in animals have any relevance to smokers. More importantly, precipitated withdrawal simply fails to occur in smokers [83-85]. This basic fact is evaded by the authors of the present report, who state: "The increase in plasma concentrations of nicotine from smoking is greater after pretreatment with mecamylamine, a nicotine receptor antagonist. The increase is probably a result of more intense puffing in an attempt to overcome the blockade of nicotine receptors [86] (p. 119)." The authors neglect to mention that the smokers in the cited study did not display the withdrawal syndrome that the report attributes to neuroadaptation, which disqualifies this study as a demonstration of precipitated withdrawal in smokers.

We should emphasize that the lack of precipitated withdrawal in smokers is a serious problem for the thesis that nicotine creates physical dependence. We are not aware of any possible pharmacological mechanism that would explain spontaneous withdrawal together with the absence of precipitated withdrawal, as in both cases nicotine does not bind to its receptor.

### Addiction and re-addiction to nicotine

Naïve animals can easily and passively be made dependent on opiates. The introduction of subcutaneous osmotic minipumps delivering 2 mg/kg/hr of morphine will result in tolerance to analgesia and a full-blown withdrawal syndrome after 48 hr [87]. With repeated exposure, humans are also likely to develop opiate dependence, and this occurs regardless of the route of administration: intravenous injection, smoking, or sniffing of heroin can all lead to dependence [88].

According to the 2010 SG report (p. 131-133) rats can be made dependent on nicotine in 7 days by continuous nicotine delivery via osmotic minipumps. What about humans? Again according to the current report (p. 157), "DiFranza and colleagues [89] concluded that, on average, the onset of an initial symptom of tobacco dependence occurred when adolescents smoked only two cigarettes once a week. Even adolescents who smoked only once or twice in their lives reported an average of 1.3 symptoms on the HONC (1.0 for males and 1.4 for females) [90]. As a cautionary note, the interpretation of the results relies on whether the HONC reflects valid symptoms of dependence". On the same page, now without a word of caution: "In one study, 19.4 percent of adolescents who smoked weekly were considered to be dependent on the basis of an analog measure from the *ICD *criteria [90]. Even less than weekly tobacco use may result in progression toward nicotine dependence. A later study found that the most susceptible youth lose autonomy over tobacco within one or two days of first inhaling from a cigarette. The appearance of tobacco withdrawal symptoms and failed attempts to stop smoking can precede daily smoking dependence, as defined by *ICD-10*, and typically appears before consumption reaches two cigarettes per day [91]".

As the "cautionary note" above hints, the research cited by the SG as demonstrating the alarming susceptibility of young smokers for developing nicotine dependence has been the target of substantial criticism [92,93] (also see linked commentaries in the same journal). Our own critique of the "hooked on nicotine" program concluded that these studies contained substantive conceptual and methodological flaws. These include an untenable and idiosyncratic definition of addiction, use of single items or of very lenient criteria for diagnosing nicotine dependence, reliance on responders' causal attributions in determining physical and mental addiction to nicotine and biased coding and interpretation of the data.

The proposition that humans are extremely susceptible to develop nicotine addiction can be tested directly by exposing naïve participants and re-exposing ex-smokers to nicotine. If adolescents can lose autonomy over tobacco within one or two days of first inhaling a cigarette, we would expect that naïve participants, and certainly ex-smokers, would show signs of nicotine addiction after prolonged exposure to nicotine. Specifically, one could use prolonged exposure to transcutaneous nicotine which, like osmotic minipumps in rats, provide significant and stable nicotine levels in plasma (see Fig. four.one in the SG report).

An experiment that could elucidate whether humans can be re-addicted to nicotine might involve a sample of never-smokers and ex-smokers. Half of each group would be exposed to nicotine-patches, delivering about 35% of the nicotine that heavy smokers would extract from their cigarettes for 12 weeks. Participants would then be followed up for 12 weeks. If the nicotine addiction thesis presented by the SG is valid, participants should develop signs of nicotine addiction. Specifically ex-smokers, who had previously learned how to cope with withdrawal and craving by smoking, would clearly be expected to resume smoking.

While such an experiment sounds ethically dubious, it has been in fact performed [94]. The reason was to examine whether transdermal nicotine would be beneficial for patients with ulcerative colitis. The experiment, using various modes of nicotine administration, was replicated several times (for review see [95]). The first experiment has special significance, because two of the co-authors (the late M.A.H. Russell and C. Feyerabend) were among the architects of the nicotine-addiction thesis. The authors summarized their results as follows: "During the trial most former smokers felt well, but the lifelong nonsmokers tolerated treatment with more difficulty. After the trial, none reported a craving for smoking, and none reported any smoking during the subsequent 12 weeks [94] (p. 814)".

## Conclusions

In its discussion of nicotine addiction, the current report of the SG presents a false picture of the current scientific knowledge in this field. The report loses credibility by uncritically endorsing research that supports its outdated model of nicotine addiction while ignoring research that refutes this model. The confirmatory bias of the report is reflected in its omission of all research on non-nicotine factors in smoking, including extensive research with de-nicotinized tobacco, in ignoring the methodological limitations and contradictory findings in regard to nicotine reinforcement in animals and in humans, and in cherry picking and ignoring evidence incompatible with its conclusions pertaining to tolerance, withdrawal and craving.

Two decades ago, Aker [96] suggested that the motivation for calling smoking an addiction was to give it a bad name. "Anything addictive is bad; if it is not addictive, it is probably not too bad. A tobacco smoking habit is bad enough, but it is even worse when one thinks of it as an addiction (p. 778)". We do not know what motivated the current report's unequivocal endorsement of the nicotine addiction thesis, but we believe that it is unlikely to be helpful to smokers. The message of the 1988 SG report proclaiming that nicotine is as addictive as heroin and cocaine was widely disseminated by scientists, physicians and the media. A 1977 study [97] reported that "About four out of five non-smokers regarded the average cigarette smoker as an addict, whereas only about half the smokers saw themselves as addicted (p. 334)". In a study published eight years later [98] only 25 out of 2,312 subjects (1%) answered the question "How addicted do you think you are to smoking?" with the answer "Not at all". Today, after more than 25 years of authoritative messages by the SG, we would not be surprised if both smokers and non-smokers view the statement "nicotine is addictive" as obviously true as "water is wet".

An addiction model inherently places control and responsibility outside the individual, so it is likely to undermine one's sense of control and self-efficacy. Indeed, smokers who believe that they are addicted perceive quitting as more difficult [99-101] and have reduced confidence in their ability to achieve complete cessation [98,102]. Moreover, these attitudes seem to act as self-fulfilling prophecies, as they are correlated with shorter duration of cessation attempts and higher relapse rates [103]. In our opinion, the SG statement on nicotine addiction is not only misleading, it will actually impede the "assault on the tobacco epidemic (p. i)" for which this report was to be the weapon.

## Competing interests

RD and HF have received fees for consulting to Imperial Tobacco Group PLC. However, all their research, including this review, is supported exclusively by academic funds.
